# Systems biology analysis of lung fibrosis-related genes in the bleomycin mouse model

**DOI:** 10.1038/s41598-021-98674-6

**Published:** 2021-09-29

**Authors:** Dmitri Toren, Hagai Yanai, Reem Abu Taha, Gabriela Bunu, Eugen Ursu, Rolf Ziesche, Robi Tacutu, Vadim E Fraifeld

**Affiliations:** 1 The Shraga Segal Department of Microbiology, Immunology and Genetics, Center for Multidisciplinary Research on Aging, Ben-Gurion University of the Negev, 8410501 Beer-Sheva, Israel; 2 Systems Biology of Aging Group, Institute of Biochemistry of the Romanian Academy, 060031 Bucharest, Romania; 3 Epigenetics and Stem Cell Unit, Translational Gerontology Branch, National Institute on Aging, NIH, Baltimore, MD 21224 USA; 4 Internal Medicine II/Pulmonology, Medical University of Vienna, 27271 Wien, Austria

**Keywords:** Respiratory tract diseases, Computational biology and bioinformatics, Data mining, Data processing, Databases, Gene regulatory networks

## Abstract

Tissue fibrosis is a major driver of pathology in aging and is involved in numerous age-related diseases. The lungs are particularly susceptible to fibrotic pathology which is currently difficult to treat. The mouse bleomycin-induced fibrosis model was developed to investigate lung fibrosis and widely used over the years. However, a systematic analysis of the accumulated results has not been performed. We undertook a comprehensive data mining and subsequent manual curation, resulting in a collection of 213 genes (available at the TiRe database, www.tiredb.org), which when manipulated had a clear impact on bleomycin-induced lung fibrosis. Our meta-analysis highlights the age component in pulmonary fibrosis and strong links of related genes with longevity. The results support the validity of the bleomycin model to human pathology and suggest the importance of a multi-target therapeutic strategy for pulmonary fibrosis treatment.

## Introduction

Tissue fibrosis is a major cause of frailty in aging and is involved in numerous age-related pathologies^1,2^. Among adult tissues, the lungs seem to be especially susceptible to age-related fibrotic pathology which is often poorly mendable^3–5^. As such, there is a great need for treatment and drug development to cope with this problem^6^. Unfortunately, experimental models of lung fibrosis are still few^7^, and it is still debatable to what extent these models adequately reflect pulmonary fibrosis in humans, in particular, idiopathic pulmonary fibrosis (IPF)^3,8,9^. The most popular experimental model, due to its ease of use, has been the mouse bleomycin-induced model^7^, in which a chemotherapeutic agent elicits a quick and robust fibrotic effect when inhaled^10^. While bleomycin does not model the disease perfectly^11,12^, it is still extremely useful for research and a widely-used model which has undoubtedly increased our understanding of fibrotic pathology^13,14^. Subsequently, a large body of data on the genetic factors that influence lung fibrosis has been accumulated based on the bleomycin-induced model. Yet, a systematic analysis of these data has not been performed to date. With this in mind, we conducted a meta-analysis of highly curated genes that have been rigorously shown to impact lung fibrosis in the bleomycin mouse model. The list containing these pulmonary fibrosis-related genes (PFRGs) is now part of the TiRe database (http://www.tiredb.org), which contains curated genetic information on wound healing and fibroproliferative processes^15^.

Apart from the collection and detailed characterization of PFRGs, we placed a special emphasis on (i) the consistency between different types of manipulations; (ii) the consistency of bleomycin data with the expression of corresponding genes in lungs of IPF patients, and (iii) the relationships between lung fibrosis-related and longevity-associated genes (LAGs).

## Results

### Lung fibrosis-related genes in the bleomycin model

We first curated a list of genes that when manipulated have been shown to have an impact on the lung fibrosis outcome in bleomycin. Overall, we found 284 entries from 196 non-redundant studies. The dominant gene-specific interventions used in bleomycin are genetic, i.e. knockout (KO) or overexpression (OE), and their targets include protein-coding as well as microRNA genes (Table 1, Supplementary Table ST1; available at the TiRe database^15^, http://www.tiredb.org). Other interventions, such as the administration of protein agonists or antagonists, or specific antibodies are less common, yet have also been carried out. Importantly, 67 genes were investigated in more than one study and by using two or more manipulations. As seen in Table 2, over 85% of the different types of manipulations, applied either in the same study or in independent studies, are fully consistent with one another with regard to their effects on bleomycin-induced lung fibrosis. The rest were either partially consistent or consistency was unclear based on available data.

**Table 1:** Gene-specific manipulation types in bleomycin-induced lung fibrosis studies.

Type of manipulation	Number of studies
Knockout	185
Overexpression	77
Overexpression and knockout	20
Protein downregulation (inhibitors, Abs)	20
Protein upregulation (agonists, external)	8
MiR knockout/knockdown	7
MiR overexpression	1
Genetic and non-genetic manipulation	22
Genetic and non-genetic (protein)	18
Genetic and epigenetic (microRNA)	3
Protein and epigenetic (microRNA)	1
Combined studies	44

For a full detailed list see Supplementary Table ST1.

**Table 2:** Consistency between the effects of genetic/protein manipulations on bleomycin-induced lung fibrosis in mice and expression of corresponding genes in human IPF.

Consistency between bleomycin model and IPF	Number of manipulations	Percentage (%)
Full	93	79.5
Partial	2	1.7
Inconsistent	10	8.5
Not clear	12	10.3
**Consistency between the effects of different manipulations**		
Full	54	85.7
Partial	7	11.1
Not clear	2	3.2

For a detailed list see Supplementary Table ST1, ST2 and ST3.

Along with the bleomycin model to test interventions, several other murine models for inducing lung fibrosis have also been used (Table 3). In all cases, when a “non-bleomycin” murine model was used for investigating the role of a given gene or protein in lung fibrosis, the effects observed were consistent with those seen in the bleomycin model (Supplementary Table ST1).

**Table 3:** Non-bleomycin models of lung fibrosis in mice.

Additional models of lung fibrosis	Number of studies
Thoracic irradiation	3
LPS	7
FITS (Fluorescein-5-isothiocyanate)	1
Crystalline silica	6
Crocidolite asbestos	3
LiCl	1
Single-walled carbon nanotubes (SWCNT)	1
MWCNT Multi-walled carbon nanotube (MWCNT)	1
Total	23

21 of 23 non-bleomycin models were used in addition to the bleomycin model in the same studies; in the case of *Lox1* and *Timp1* genes, only non-bleomycin models were used.

Overall, the curated list of PFRGs consists of 216 unique (non-redundant) genes that were examined regarding their role in bleomycin-induced lung fibrosis in mice (Supplementary Table ST1). Of them, only 3 genes (*Ptgs1*, *Rps6kb1*, *Rps6kb2*) did not show a definite impact. The PFRGs that displayed an effect could be further divided into two major groups: anti-fibrotic and pro-fibrotic. We considered a gene as anti-fibrotic if its upregulation reduced fibrosis and/or its downregulation had an opposite effect. Conversely, if upregulation promoted lung fibrosis and/or downregulation reduced fibrosis, the gene was considered pro-fibrotic. In our dataset, the number of anti-fibrotic genes was approximately equal to that of pro-fibrotic genes: 43.5% of genes displayed a clear anti-fibrotic activity, 50% of genes displayed pro-fibrotic activity, and 6.5% of genes showed inconsistent results (both pro- and anti-fibrotic). Remarkably, we noted a high consistency (~ 80%) between the effects of genetic manipulations of PFRGs in bleomycin-induced lung fibrosis in mice and the expression of their human orthologs in the lungs of patients with IPF (Table 2).

### Evolutionary conservation of pulmonary fibrosis-related genes

**Figure 1:**
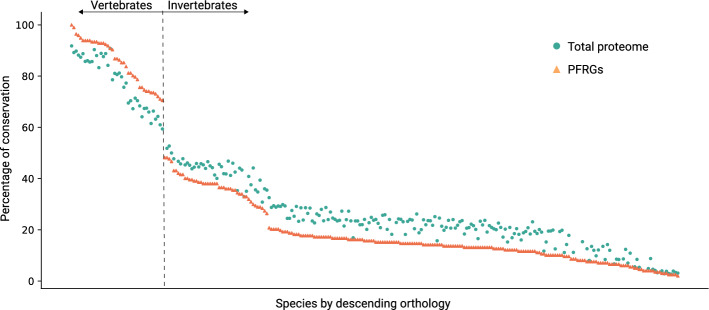
Evolutionary conservation of pulmonary fibrosis-related genes (PFRGs). The graph summarizes the percentages of orthologs between humans and given species. Each dot corresponds to a single species from InParanoid (in descending order by the percentage of orthology). A total of 268 species from all kingdoms of life are presented. Orange triangles stand for the percentage of orthologs for PFRGs (n = 195); green circles stand for the percentage for the entire human genome (n = 20,297). The difference in ortholog percentage between the entire genome and PFRGs is significant for the vast majority of species: Chi-square (χ^2^) goodness of fit, *p* < 0.05.

PFRGs were studied in mice but are most interesting for their potential impact on humans. Consequently, we also evaluated their evolutionary conservation in more depth. For this purpose, we extracted the PFRG orthologs for all species available in the InParanoid database^16^; http://inparanoid.sbc.su.se/). Some of the PFRGs (n = 18) were not represented in InParanoid and the analysis was performed on 195 genes only. As seen in Fig. 1, PFRGs are differentially conserved among vertebrates and invertebrates: they are over-represented in vertebrates and under-represented in invertebrates.

### Enrichment analysis

Next, we looked to see whether the PFRG list is enriched for certain processes and pathways that dominate in pulmonary fibrosis. We found that PFRGs are enriched for processes such as regulation of proliferation, inflammation and immune functions, and aging processes (Table 4). Interestingly, there are marked enrichment differences between pro-fibrotic and anti-fibrotic genes: (i) While pro-fibrotic genes are enriched for genes that positively regulate proliferation, anti-fibrotic genes are composed of those that negatively regulate it; (ii) pro-fibrotic genes are also enriched for pathways that relate to inflammation and immune function while anti-fibrotic genes are not.

**Table 4:** Processes and pathways enriched in pulmonary fibrosis-related genes (PFRGs).

All PFRGs	Pro-fibrotic PFRGs	Anti-fibrotic PFRGs
*Regulation of proliferation*	*Positive regulation of proliferation*	*Negative regulation of proliferation*
*Cytokine signaling*	*Cytokine signaling*	
*Inflammation*	*Inflammation*	
*Immune function*	*Immune function*	
*Cancer*	*Cancer*	*Cancer*
*Reaction to pathogen*	*Reaction to pathogen*	
*Oxygen homeostasis*	*Oxygen homeostasis*	
*MAPK signaling pathway*		*MAPK signaling pathway*
*TNF signaling pathway*	*TNF signaling pathway*	
*Jak-STAT signaling pathway*	*Jak-STAT signaling pathway*	
Asthma		
Aging		
	Insulin resistance	
	Stress response	
		Estrogen signaling pathway
		Response to mechanical stimulus
	PI3K-Akt signaling pathway	
	VEGF signaling pathway	
	FoxO signaling pathway	
	Apoptosis	

For a full enrichment analysis see Supplementary Table ST4. Italicised areas that are common to all, pro-fibrotic, and anti-fibrotic PFRGs.

**Figure 2:**
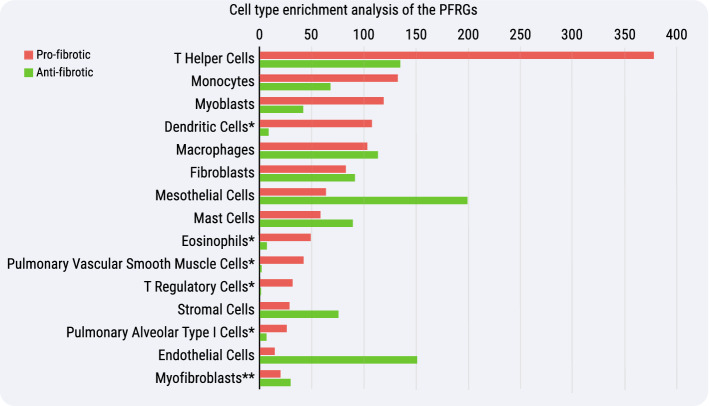
Cell type enrichment analysis of the PFRGs. Pro- and anti-fibrotic genes were tested for expression enrichment in specific cell types. Analysis was performed against the PanglaoDB database on the Enrichr platform. Presented are the main enriched cell types for both lists and their combined enrichment score (see methods) for pro-fibrotic (red) and anti-fibrotic (green). *Non-significant enrichment in anti-fibrotic genes (*p* > 0.05) and **Non-significant enrichment in pro-fibrotic genes (*p* > 0.05).

An important question is whether there is any association between pro-fibrotic or anti-fibrotic genes and specific cell types. Since the vast majority of data on gene expression in pulmonary fibrosis were obtained for the whole lung tissue, a direct answer to this question is at the moment impossible. Nevertheless, we took advantage of the Enrichr tools to estimate what tissues and cell types are more likely to be associated, if any, with PFRGs. The Human Gene Atlas database^17^ (http://biogps.org) analysis revealed that our set of anti-fibrotic genes is more likely to be expressed in lungs, while the pro-fibrotic genes are more likely to be expressed in vessels, lungs, and blood. We further used the PanglaoDB database^18^ (http://panglaodb.se) for determining cell types associated with PFRGs. As seen in Fig. 2, the pro-fibrotic genes are mostly associated with immune cells. Apart from immune cells, the anti-fibrotic genes are mostly associated with connective tissue and endothelial cells.

### Functional module analysis

**Figure 3:**
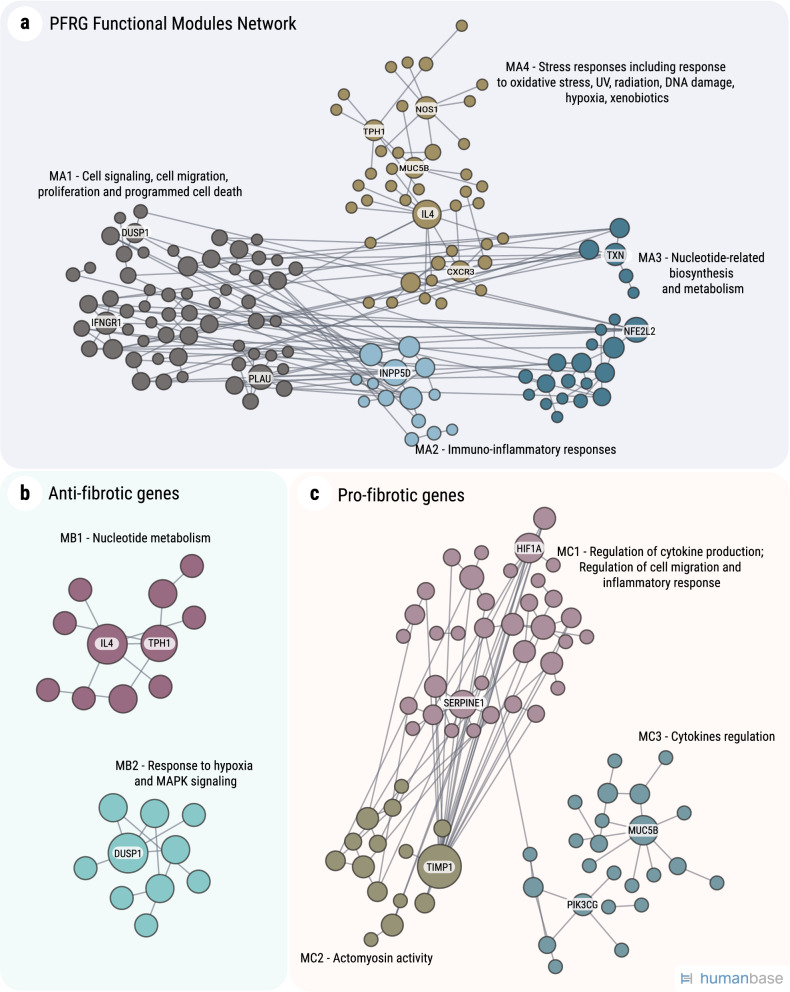
Functional module network of the pulmonary fibrosis-related genes (PFRGs) in lung tissue. The network was built for the lung tissue, using HumanBase, with the human orthologs of PFRGs as input. The interaction network is built using the closest gene neighbors and then clustered based on enrichment in GO categories. (**a**) All PFRGs. (**b**) Anti-fibrotic genes. (**c**) Pro-fibrotic genes. The networks were generated with HumanBase online tool (https://hb.flatironinstitute.org).

To further understand the characteristics of the PFRG network, we conducted a functional module analysis using the HumanBase online tool^19^ (https://hb.flatironinstitute.org). This tool allows for identifying functional modules in a gene-set in a manner specific to a given tissue (in this case, lungs). The analysis revealed six functional modules formed by PFRGs and their immediate partners (Fig. 3a and Supplementary Table ST5). These include: (i) a cluster encompassing cell signaling, cell migration, proliferation and programmed cell death (MA1, 643 GO terms; *p* < 0.05); (ii) a cluster of immuno-inflammatory responses (MA2, 177 GO terms; *p* < 0.05); (iii) a cluster pertaining to nucleotide-related biosynthesis and metabolism (MA3, 159 GO term; *p* < 0.05); and (iv) a cluster focused on stress responses including response to oxidative stress, UV, radiation, DNA damage, hypoxia, xenobiotics (MA4, 215 GO terms; *p* < 0.05).

For anti-fibrotic genes, there are two functional modules that include at least ten genes each. The first cluster (MB1, 65 GO terms; *p* < 0.05) includes mostly nucleotide metabolism, and the second module (MB2, 137 GO terms; *p* < 0.05) encompasses response to hypoxia and MAPK signaling (Fig. 3b). The list of pro-fibrotic genes includes three functional modules: the first one, (MC1, 356 GO terms; *p* < 0.05) covers regulation of cytokine production, regulation of cell migration and inflammatory response; the second module (MC2, 92 GO terms; *p* < 0.05) is in relation to actomyosin activity and the third module (MC3, 163 GO terms; *p* < 0.05) includes cytokine regulation.

### Network analysis

**Figure 4:**
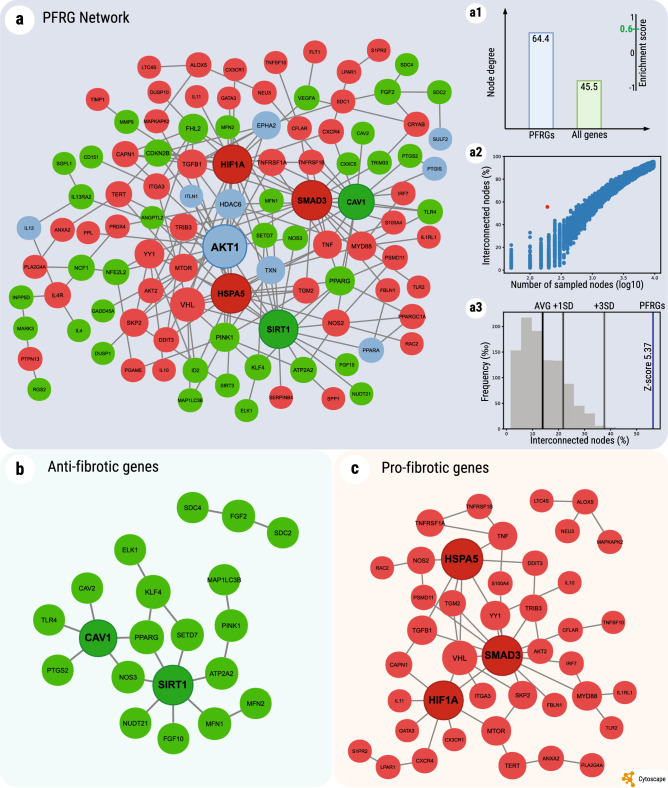
Network interactions between the human orthologs of pulmonary fibrosis-related genes (PFRGs). (**a**) Protein–protein interaction network of PFRGs. The network was constructed using the BioGRID database. Depicted is the largest continuous component of the pulmonary fibrosis network (107 nodes). Green nodes represent anti-fibrotic genes (N = 41), red are pro-fibrotic genes (N = 55), and blue nodes are genes with an unclear (both pro- and anti-) effect (N = 11). Pulmonary fibrosis hubs, the topmost 5% connected genes in the network, are highlighted with bold text and a black border. (**a1**) PFRGs (blue bar) are significantly more connected than random interactome genes (green bar), with a higher average number of protein–protein interactions (64.4 for PFRGs vs. 45.5 for all genes) and a GSEA-based enrichment score of 0.6 for degree connectivity (*p* < 0.05). (**a2**) The observed interconnectivity of PFRGs in the interactome, depicted by the red dot in the scatter plot, can be compared to the percentage of interconnected nodes (on the Y-axis), found in the largest continuous component, for randomly sampled node sets. The plot shows the sampling of subsets of random interactome nodes, of various sizes (represented in a log10 scale on the X-axis, from 50 to 17,600 nodes), for which the interconnectivity was computed 100 times. (**a3**) The interconnectivity of the PFRG network (blue line), compared to the histogram of frequencies of interconnectivity, per one thousand random samples of the same geneset size (Y-axis). PFRG interconnectivity (56.31%) is significantly larger than expected, with a Z-score of 5.37 (distribution average 13.9%, SD: 7.9%). (**b**) Anti-fibrotic genes in the PFRG network form a continuous subnetwork (26 out of 107, 24.3%). (**c**) Pro-fibrotic genes in the PFRG network form a continuous subnetwork (42 out of 107, 39.3%). The networks were generated by Cytoscape 3.8.0. (https://www.cytoscape.org). The panels **a1**–**a3** were generated using a custom R script developed in-house.

To further understand the features of PFRGs and how they relate to each other, we performed a protein–protein interaction (PPI) network analysis on the protein products of PFRGs. As the human proteome is more comprehensively mapped^20^, the analysis was done on the human orthologs of the mouse PFRGs. The analysis shows that PFRGs are highly connected in the interactome (Fig. 4, panel a1) and strongly interact among themselves, forming a large, directly connected component of 107 nodes (56.3% of all PFRGs). This interconnectivity, i.e. the fraction of genes/nodes forming the largest connected subnetwork from a certain gene set, is higher for PFRGs than expected by chance (Fig. 4, panel a2). The statistical significance of this observation was validated by comparing it with the results from random gene set samples of equal size (Fig. 4, panel a3). Considering this, it is not surprising that several genes in the network are also important hubs, i.e., display very high connectivity with other genes in the network. For example, the topmost 5% network hubs (with 13–21 PFRG interactions and > 150 interactome PPIs), depicted in bold in Fig. 4, include AKT1 (a regulator of mTOR signaling), CAV1, SIRT1 (epigenetic regulator), HSPA5 (heat shock protein), SMAD3 (plays a role in TGFβ signaling), and HIF1A (response to oxygen). Of note, the CAV1 gene was found to be a large hub in both the functional anti-fibrotic modules (Fig. 3b, MB) and in the PFRG PPI network (Fig. 4a, b). Additionally, the two network hubs HIF1A, which is linked to aging and response to hypoxia^21^, and SMAD3, known as an important player in wound healing and an anti-longevity gene^15,21^, were also found in the pro-fibrotic module (Fig. 3c, MC1). Another hub of the PFRG network, the AKT1 protein (Fig. 4a) is well-known for its anti-apoptotic activity and has been linked to cellular senescence^22^.

### Links between pulmonary fibrosis genes and longevity

**Figure 5:**
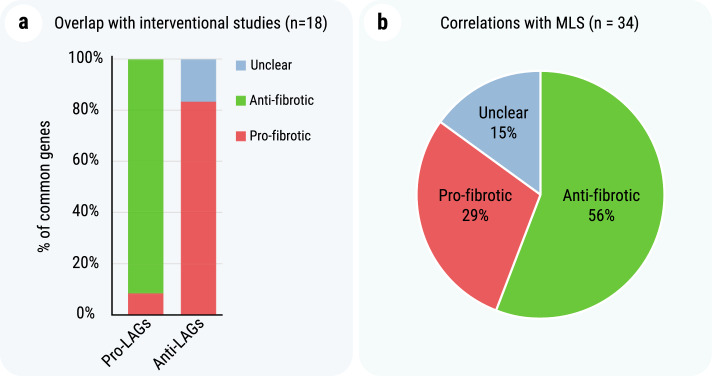
Links between pulmonary fibrosis-related genes (PFRGs) and longevity. (**a**) Distribution of longevity-associated genes (LAGs) by their role in bleomycin-induced lung fibrosis in mice. 18 genes form the overlap between known LAGs and PFRGs. Among 12 pro-LAGs, 11 have an anti-fibrotic effect, while at least five out of the six anti-LAGs are pro-fibrotic. (**b**) Linear models of PFRG expression in lung tissue relative to maximum lifespan (MLS) identified 34 gene correlations (out of 90 PFRGs with orthologs in all considered species), for both pro- and anti-fibrotic genes (R^2^ between 0.18 and 0.63).

Interestingly, many of the enriched categories in the functional modules analysis (Fig. 3., e.g. MA2) are also relevant to aging, while at the same time, many genes from MA3 (Fig. 3) are in fact longevity-associated genes (LAGs). This prompted the following question: Do genes that influence lung fibrosis have any impact on longevity in mice? To get insight into this issue, we compared the list of PFRGs with the list of LAGs from GenAge, which were reported to affect the lifespan of mice^21,23^. The comparison yielded 18 genetic mouse models of extended lifespan (longevity phenotype) or reduced lifespan (premature aging phenotype), which were also tested for their role in bleomycin-induced pulmonary fibrosis. The results summarized in Fig. 5a and Table 5, clearly show that pro-longevity genetic manipulations also reduce pulmonary fibrosis, while anti-longevity genetic manipulations have the opposite effect. That is, pro-longevity genes tend to be associated with anti-fibrosis (11 out of 12 pro-LAGs are anti-fibrotic), while anti-longevity genes with pro-fibrosis (5 out of 6 anti-LAGs are clearly pro-fibrotic with an additional anti-LAG showing both anti- and pro-fibrotic effects; Fisher's exact test, *p* = 0.001).

**Table 5:** Genes/proteins that when manipulated had an effect on both longevity and lung fibrosis in mice.

Targeted gene/protein	Impact on longevity	Impact on lung fibrosis
*Akt1*	Anti-Longevity	Pro-fibrotic
*Akt2*	Anti-Longevity	Pro-fibrotic
*Cav1*	Pro-Longevity	Anti-fibrotic
Fgf2	Pro-Longevity	Anti-fibrotic
*Foxm1*	Pro-Longevity	Anti-fibrotic
*Kl (Klotho)*	Pro-Longevity	Anti-fibrotic
*Mtor*	Anti-Longevity	Pro-fibrotic
*Nos3*	Pro-Longevity	Anti-fibrotic
*Parp1*	Anti-Longevity	Pro-fibrotic
*Plau*	Pro-Longevity	Anti-fibrotic
*Pparg*	Pro-Longevity	Anti-fibrotic
*Rps6kb1*	Anti-Longevity	unclear
*Serpine1 (PAI-1)*	Anti-Longevity	Pro-fibrotic
*Sirt1*	Pro-Longevity	Anti-fibrotic
*Sod3*	Pro-Longevity	Anti-fibrotic
*Tert*	Pro-Longevity*	Pro-fibrotic
*Txn1*	Pro-Longevity	Anti-fibrotic
*Zmpste24*	Pro-Longevity	Anti-fibrotic (in old age)

*Tert—The final impact on longevity is not entirely clear (for details please see the HAGR—GenAge database for details; https://genomics.senescence.info/genes/index.html). For detailed description, see Suppl. Table ST8.

The analysis above suggests that the anti- and pro-fibrotic genes have evolved in tight relation to the anti- and pro-longevity genes, with many of these genes even having direct roles in both lifespan determination and fibrosis development. To further test this notion, we decided to investigate to what extent the gene expression levels of PFRGs correlate with maximum lifespan (MLS). For this we used the tools developed in our lab and collected data, comprising 28 samples from 14 mammalian species, analyzed in our recent cross-species lifespan study^24^. The analysis showed that the expression of many PFRGs (n = 34) correlates with MLS in mammals (Fig. 5b; Supplementary Table ST6 and ST7). The number of these MLS-associated genes is 2.34 times more than expected by chance (Fisher's exact test, *p* = 6.4E−05), with R^2^ being even greater than 0.6 for some of the correlations, overall suggesting that many fibrosis-related genes might have had a role in the determination of MLS as well.

## Discussion

Although many animal models have thus far been established for investigating IPF^7^, the bleomycin model, despite its limitations and disadvantages, is the most widely used and generally viewed as the standard in modeling pulmonary fibrosis^25^. In this study, we collected a list of over 200 genes (PFRGs) influencing the course and/or outcome of bleomycin-induced pulmonary fibrosis, and performed a comprehensive analysis on their role in the bleomycin mouse model. The PFRGs are currently available in the TiRe database, which contains curated genetic information on wound healing and fibroproliferative processes^15^ (hosted at http://www.tiredb.org). The results of the analysis pointed out several important findings:

(i) A high consistency between the different types of genetic and non-genetic manipulations in their effects on bleomycin-induced lung fibrosis. When the same manipulation was used in different studies or different manipulations were used in the same study, the consistency between results supports their reliability. In interpreting and evaluating these results, it should be considered that the list of PFRGs relies mostly on studies that employ loss-of-function interventions (Table 1) and thus might cover only a part of the molecular mechanisms involved in fibrosis. Still, even if the collected PFRGs do not encompass the whole picture of fibrosis, they seem to provide a coherent image as they form a highly interconnected PPI network.

Another important point is that PFRGs might be involved in a more general response, i.e. tissue repair after injury. In particular, we compared PFRGs with skin wound healing-related genes^15^ and found a significant overlap of over 20% (*p* < 0.001). This observation suggests that fibrosis and wound healing have much in common and that PFRGs are not exclusively associated with lung fibrosis but rather many of them are involved in a more general response. Yet, it seems the relationship between these processes is more complex than a simple “accelerated/slower wound healing—reduced/promoted fibrosis”. These relationships are an excellent point for future investigations.

(ii) PFRGs are overall enriched for regulation of cellular proliferation, inflammation and immune functions, and aging-related processes, with a prominent difference between anti- and pro-fibrotic genes. That is, when pro- and anti-fibrotic genes were analyzed separately, they displayed definite enrichment patterns that were distinct from one another. We found that the pro-fibrotic genes are dominated by positive regulation of cellular proliferation, inflammatory processes and immune responses, including related processes and pathways such as Cytokine signaling, Jak-STAT signaling pathway, TNF signaling pathway, or Reaction to pathogen. These findings are not unexpected when considering that fibrosis is a wound healing response gone awry, which would most likely be the case for fibrosis of any tissue. The above indirectly highlights the role of immunity and inflammatory responses in the induction and development of pulmonary fibrosis^9^, a conclusion that is also supported by the results of our functional module analysis. Remarkably, pro-fibrotic genes are specifically enriched in processes and pathways closely linked to aging, such as the insulin-FoxO signaling pathway^26^, PI3K-Akt signaling pathway^22^, etc. Not surprisingly, PFRGs and particularly pro-fibrotic genes are enriched for the Oxygen homeostasis and Stress response categories. This is in line with our previous finding of high resistance to oxidative-stress-induced cytotoxicity in lung fibroblasts from IPF patients^27^. Huang et al.^28^ have also shown that fibroblasts from the lungs of bleomycin-treated old mice, displayed a stronger fibrotic response and were more resistant to H_2_O_2_-induced apoptosis than those from the young. In the long run, this may result in the accumulation of damaged/senescent cells that would otherwise be eliminated, as was actually observed in vivo by Hecker et al.^29^. In that regard, we were surprised to find that cellular senescence per se was not one of the terms found in the enrichment analysis. However, many of the pathways that we found can potentially converge to it (e.g. regulation of proliferation, cancer, inflammation, response to stress, etc.), thus supporting the idea that cellular senescence may indeed play an important role in lung fibrosis^6,27,30,31^. The results of enrichment analysis are further strengthened by the functional module network analysis in which at least two modules are enriched for processes related to the response to oxidative stress and hypoxia (see Fig. 3 and Supplementary Table ST5). This could be specifically relevant to lung vs other tissues, because of the high oxygen environment^32,33^. In contrast to pro-fibrotic genes, anti-fibrotic genes were found to be enriched for negative regulation of cell proliferation, MAPK signaling pathway and response to mechanical stimulus. Of note, the lungs experience ongoing mechanical stress and areas with a higher pressure are more prone to fibrotic changes^34^. With this in mind, our previous finding of actin-organization aberrations in IPF fibroblasts^27^ may highlight the critical role of the response to mechanical stress in lung integrity and functionality. Interestingly, PFRGs are most likely to be expressed in immune cells and connective tissues cells, pro-fibrotic genes being mainly associated with immune cells, whereas anti- fibrotic genes with connective tissues, pulmonary vascular smooth muscle and endothelial cells (Fig. 2).
(iii) The high consistency between the expression of PFRGs in the mouse model of bleomycin-induced lung fibrosis and the expression of their human orthologs in the lungs of IPF patients indicates that, despite its disadvantages^11,12^, the bleomycin model is still highly relevant for the study of human lung fibrosis^10,25,35^. In particular, the human orthologs of murine PFRGs could be the targets for therapeutic interventions. To some extent, it is also supported by our findings that PFRGs are highly conserved among vertebrates but much less in invertebrate species. This implies that many PFRGs are a relatively recent acquisition in the course of evolution and that the genetic basis of pulmonary fibrosis may have a common platform among vertebrates. Yet, the targets for manipulation might have been, to some extent, selected a priori based on indications for their potential involvement in IPF. If so, it may cause a bias for the PFRG list.
(iv) Our network-based analysis clearly showed that PFRGs are highly interconnected and hence interacting, thus significantly reducing the odds to treat pulmonary fibrosis by targeting a single gene^36^. In other words, it means that a multi-target therapy approach would definitely be preferable.
(v) Comparing the mouse PFRGs and longevity-associated genes (LAGs) brought another remarkable finding: pro-longevity genes are dominated by anti-fibrotic genes, whereas the anti-longevity genes are dominated by pro-fibrotic genes. Congruent with this finding is the observation that the functional modules for anti-fibrotic genes contain pro-LAGs but no anti-LAGs (Fig. 3b). Conversely, the modules for pro-fibrotic genes include mostly anti-LAGs (Fig. 3c). That is, the anti-fibrotic genes are associated with lifespan extension while the pro-fibrotic ones are associated with premature aging. Both findings support the notion that pulmonary fibrosis is a disease of aging. It would be worth mentioning that many age-related processes are linked to signaling pathways crucially involved in organ regeneration/repair (though on various levels of the metabolic hierarchy)^37^. They concern structural and functional differentiation as well as de-differentiation of organ tissue, chronic inflammation, and energy supply (or more precisely, its depletion). However, at this point, we should still keep in mind that our analysis predominantly represents pulmonary fibrosis induced by bleomycin and does not necessarily reflect (in both quality and quantity) the entirety of the most frequent and threatening form of age-related human lung fibrosis, IPF. In addition, it is difficult to assess the “chicken and egg” dilemma at this point. That is, we cannot definitely state whether manipulation of these genes affects fibrosis primarily, which in turn drives tissue aging, or the more likely case, that these genes are integral to important processes that affect both aging and fibrosis in parallel.

It should be noted that the vast majority of the bleomycin studies were done using young mice, whereas the logic of lung fibrosis, and in particular IPF, requires including aged animals in the study. Thus far, these studies are sporadic. As an example, young WT and Zmpste24-deficient progeroid mice developed a similar fibrotic response to BLM. In contrast, old WT mice but not old Zmpste24-deficient mice developed severe lung fibrosis^38^. Unexpected protection of Zmpste24-null old lungs against BLM was apparently attributed to the upregulation of several extracellular matrix-related miRNAs (miR23a, 27a, 29a, 145a), thus resulting in downregulation of targeted profibrotic pathways of TGF-β/SMAD3/NF-κB and Wnt3a/β-catenin signaling axes. Of note, similar age-dependent responses were observed when the rate of skin wound healing was investigated in longevity/premature-aging phenotypes^15,39,40^. Nevertheless, the mouse BML model, even when used in young animals alone, appears to be a valuable tool for investigating lung fibrosis.

Finally, we would like to stress again that, based on our analysis, a multi-target therapy of pulmonary fibrosis should become the major strategy. Furthermore, although the collected list of PFRGs is quite extensive, the application of novel techniques, such as CRISPR and modified RNA, would be an important point for future investigations. It is also worthwhile to extend the experimental studies by including aged animals.

## Methods

### Data sources

The list of the PFRGs was compiled from peer-reviewed literature and the extracted data were manually curated by the authors. The data were organized in a tabular format (and is available as an Excel table in Supplementary Table ST1, ST2 and ST3), and the curation process focused on the extraction of the following characteristics: targeted gene/protein, Ensembl ID, mouse strain, manipulation type, gender, age, dose/route of administration, regimen, main effects, pro- or anti-fibrotic effect, other effects, and relevant references. In order for a paper to be considered for the analysis, each article had to meet the following criteria: (1) To use the mouse model of BML-induced lung fibrosis, with sufficient fibrosis markers and follow-up description; (2) To contain the data on genetic or protein manipulations resulted in a significant promotion or suppression of BML-induced lung fibrosis. In addition, for comparative analysis between BLM-induced lung fibrosis and IPF, only the IPF papers with the expression of the gene of interest (i.e. genetically manipulated in BLM-induced lung fibrosis) were included.

### Evolutionary conservation

The evolutionary conservation analysis was performed using Python scripts developed in our lab, which automatically extract and analyze data from the InParanoid database^16^, version 8 (http://inparanoid.sbc.su.se/cgi-bin/index.cgi). For each mouse gene, the presence or absence of orthologs across 268 proteomes (all species available in InParanoid) was assessed and the overall evolutionary conservation was defined as the percentage of species in which at least one ortholog exists. The evaluation of orthology in InParanoid was performed with a threshold for the inparalog score of 1.0 (highest stringency). All comparisons were statistically significant unless otherwise mentioned (Chi-Square χ^2^ test; *p* < 0.05). For the network analysis, the human orthologs for mouse PFRGs were computed using a similar method (InParanoid 8 inparalogs with scores of 1.0).

### Enrichment analysis

Enrichment analysis of PFRGs and related pathways was performed using the DAVID Bioinformatics Resources tool^41^, version 6.8, https://david.ncifcrf.gov. As the data on human genes and proteins is the most abundant, the human orthologs of PFRGs defined in mice were used for the analysis. Statistical significance of enrichment was evaluated using the default parameters set in DAVID. Cell type enrichment analysis of the PFRGs was performed against the PanglaoDB database^18^ (https://panglaodb.se) using the Enrichr platform^42^ (https://maayanlab.cloud/Enrichr/). The presented combined score is the multiplication of the natural logarithm of p-value (Fisher exact test) and the z-score of the deviation from the expected rank (for more details, please see: https://maayanlab.cloud/Enrichr/). To determine the most likely tissue, we used the Human Gene Atlas database^17^ (http://biogps.org/).

### Protein–protein interaction network

Protein–protein interaction (PPI) data were taken from the BioGRID database^20^, http://thebiogrid.org, human interactome, Build 3.5.188. The PPI network construction and analysis were performed using Cytoscape^43^, http://www.cytoscape.org, version 3.8.0. Prior to any network analyses, genetic interactions, self-loops, duplicate edges and interactions with proteins from other species were removed from the interactome, and the remaining network was used as a control. The interconnectivity was computed as the fraction of nodes in the largest connected component out of the input gene set, by using the breadth-first search algorithm. Modeling the relationship between node subset size and interconnectivity in the human interactome (Fig. 4, panel a2) was carried out by randomly sampling subsets of nodes in the interactome, with a sample size varying from 50 to 17,600 nodes (step of 50). In this case, sampling was performed 100 times for each subset size. To evaluate the statistical significance of the observed network interconnectivity, random sampling of 190 nodes from the BioGRID network was performed 1000 times. The enrichment score for the degree of PFRGs in the PPI network was computed using the GSEA method^44^.

### Functional module analysis

The construction of a network with functional modules for FPRGs was carried out using the HumanBase tool^45^, https://hb.flatironinstitute.org, with a minimum module size set to 10 genes. Briefly, HumanBase provides the possibility to identify, at the tissue level, functional modules containing genes and their interaction partners which specifically work together, by grouping them into clusters of relevant biological processes. HumanBase detects modules of genes from tissue-specific functional association gene networks built by integrating vast omics datasets and associates terms (e.g. processes, pathways) to the detected modules based on overrepresentation.

### Linear models linking longevity and gene expression

The linear longevity models for the PFRGs dataset included 14 species (Homo sapiens not included) with reported maximum lifespan and a total of 28 lung transcriptome samples (Supplementary Table ST6). 8205 genes were selected based on the orthology relationships and lung expression. Raw gene expression cross-species data was extracted from public archives and reanalyzed with an internal pipeline described elsewhere^24^. The orthology relationships were obtained from the 99th release of Ensembl Compara Database^46^; https://doi.org/10.1093/database/bav096. 90 of the PFRGs were considered for analysis based on orthology relationships among the 15 species, as described in Kulaga et al.^24^. Maximum lifespan data were extracted from the AnAge database^21^; https://genomics.senescence.info/species. The analysis was performed using python scripts developed in our lab using several packages including Pandas, Seaborn, and Statsmodels. The analysis includes species with good quality assemblies and annotations and genes with orthologs in the selected species. For the evaluation of statistical significance (*p* < 0.05), the adjusted p-values with Benjamini–Hochberg correction were used. The models were defined and fitted using the “statsmodels” Python module^47^.

## Supplementary Information

Supplementary Table 1.

Supplementary Table 2.

Supplementary Table 3.

Supplementary Table 4.

Supplementary Table 5.

Supplementary Table 6.

Supplementary Table 7.

Supplementary Table 8.
